# Functional genomics in stem cell models: considerations and applications

**DOI:** 10.3389/fcell.2023.1236553

**Published:** 2023-07-24

**Authors:** Kaivalya Shevade, Sailaja Peddada, Karl Mader, Laralynne Przybyla

**Affiliations:** ^1^ Laboratory for Genomics Research, San Francisco, CA, United States; ^2^ Department of Biochemistry and Biophysics, University of California, San Francisco, San Francisco, CA, United States

**Keywords:** iPSC (induced pluripotent stem cell), human disease, CRISPR screening, functional genomics, iPSC-derived models, drug discovery

## Abstract

Protocols to differentiate human pluripotent stem cells have advanced in terms of cell type specificity and tissue-level complexity over the past 2 decades, which has facilitated human disease modeling in the most relevant cell types. The ability to generate induced PSCs (iPSCs) from patients further enables the study of disease mutations in an appropriate cellular context to reveal the mechanisms that underlie disease etiology and progression. As iPSC-derived disease models have improved in robustness and scale, they have also been adopted more widely for use in drug screens to discover new therapies and therapeutic targets. Advancement in genome editing technologies, in particular the discovery of CRISPR-Cas9, has further allowed for rapid development of iPSCs containing disease-causing mutations. CRISPR-Cas9 technologies have now evolved beyond creating single gene edits, aided by the fusion of inhibitory (CRISPRi) or activation (CRISPRa) domains to a catalytically dead Cas9 protein, enabling inhibition or activation of endogenous gene loci. These tools have been used in CRISPR knockout, CRISPRi, or CRISPRa screens to identify genetic modifiers that synergize or antagonize with disease mutations in a systematic and unbiased manner, resulting in identification of disease mechanisms and discovery of new therapeutic targets to accelerate drug discovery research. However, many technical challenges remain when applying large-scale functional genomics approaches to differentiated PSC populations. Here we review current technologies in the field of iPSC disease modeling and CRISPR-based functional genomics screens and practical considerations for implementation across a range of modalities, applications, and disease areas, as well as explore CRISPR screens that have been performed in iPSC models to-date and the insights and therapies these screens have produced.

## Introduction

As CRISPR-based genetic screening techniques become more commonplace and stem cell technologies advance to recapitulate disease relevant biology with increasing level of detail, there is a need to advance implementation of human disease-relevant models in high-throughput screening platforms, which we aim to facilitate by outlining the potential use cases and advantages and disadvantages of these platforms, the practical considerations involved, and existing applications for these technologies.

Pluripotent stem cells (PSCs) hold great potential in disease modeling due to their ability to differentiate into all cell types in the human body (Romito and Cobellis, 2016). There currently are dozens of well-characterized differentiation protocols to make a variety of cell types and this list is expanding rapidly (Pandya et al, 2017; Mahajani et al, 2019; Lyra-Leite et al, 2022). The discovery that induced pluripotent stem cells (iPSCs) can be generated by reverting fully differentiated cells to a stem-like state has further expanded disease modeling capability (Takahashi and Yamanaka, 2006) and increased access to human PSCs (Moradi et al, 2019). These iPSCs carry with them the genetic background of the donor cell type, which allows for the study of disease in a cell-type and genetically relevant context (Li L. et al, 2018). Additionally, advancements in gene editing (Jinek et al, 2012; Rees and Liu, 2018) and 3D organoid culture (Dutta et al, 2017), place iPSC-derived cell models in a unique position to study disease in a physiologically relevant context.

With the advent of CRISPR/Cas9 systems in 2012, genome editing and functional genomics screening platforms have likewise seen a rapid technological progression. While the first iteration of CRISPR-based screens relied on targeted single base insertions or deletions (CRISPR knockout), subsequent systems such as CRISPR interference and activation (CRISPRi/a) offer the ability to modulate endogenous gene expression. These CRISPR systems rely on the fusion of functional protein domains to catalytically inactive dCas9, and when run reciprocally in gain- and loss-of-function screens, for example, can identify more complex gene interactions than a knockout screen alone (Gilbert et al, 2014). There are also many CRISPR systems on the horizon that address different aspects of biology including DNA-methylation based gene silencing (Nuñez et al, 2021), direct targeting of RNA (Abudayyeh et al, 2017), multiplex gene targeting and base editing (Koblan et al, 2018; Porto et al, 2020).

**FIGURE 1:**
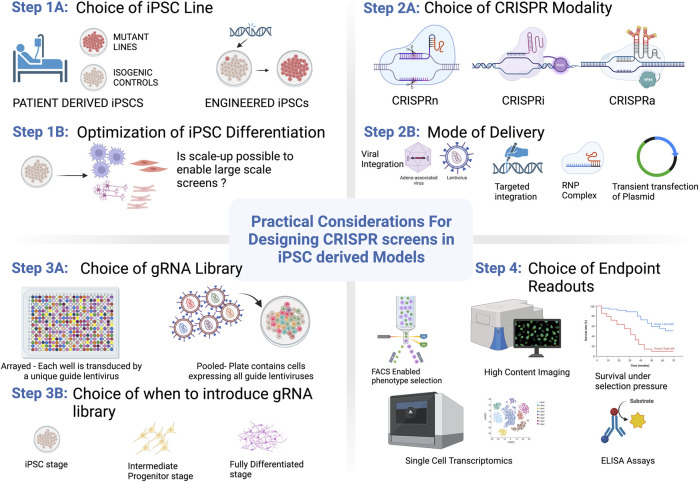
Practical considerations for getting started with CRISPR/Cas9 screening in PSCs. Options and considerations for each step are described in more detail throughout the text. Figure created with BioRender.com.

As the fields of iPSC modeling and CRISPR genome editing progress it becomes increasingly clear that harnessing the power of CRISPR-based screening in complex iPSC-derived models offers the potential to elucidate new and exciting biology in a high-throughput manner. Though the applications and scope of functional genomics screening platforms that utilize PSC-derived cells have been steadily increasing in recent years, several challenges remain that make these techniques more difficult to access than screening in standard cell line models. In this review we discuss the advantages and limitations of disease modeling in PSCs, including the ability to utilize them in more sophisticated model systems, considerations around genetic variation, and publicly available repositories with disease-relevant iPSC lines. Challenges also remain in terms of identifying, optimizing, and implementing a relevant screening assay for a particular biological question. To address these challenges, we discuss types of screening modalities and describe assay and readout capabilities that are compatible with iPSC-models, including strategies to overcome some of the current challenges in the field. In doing so, we outline the practical aspects to consider when designing a CRISPR screen in iPSCs as broken down by key steps within each category (Summarized in Figure 1). To highlight examples in the literature, we describe high-throughput screens that have been run in iPSC-derived models including key discoveries that have advanced our understanding of human disease biology. Finally, taking into account these considerations we discuss the future directions for iPSC-derived cells in the context of CRISPR screening.

## Advantages of using PSC-derived cells

**FIGURE 2:**
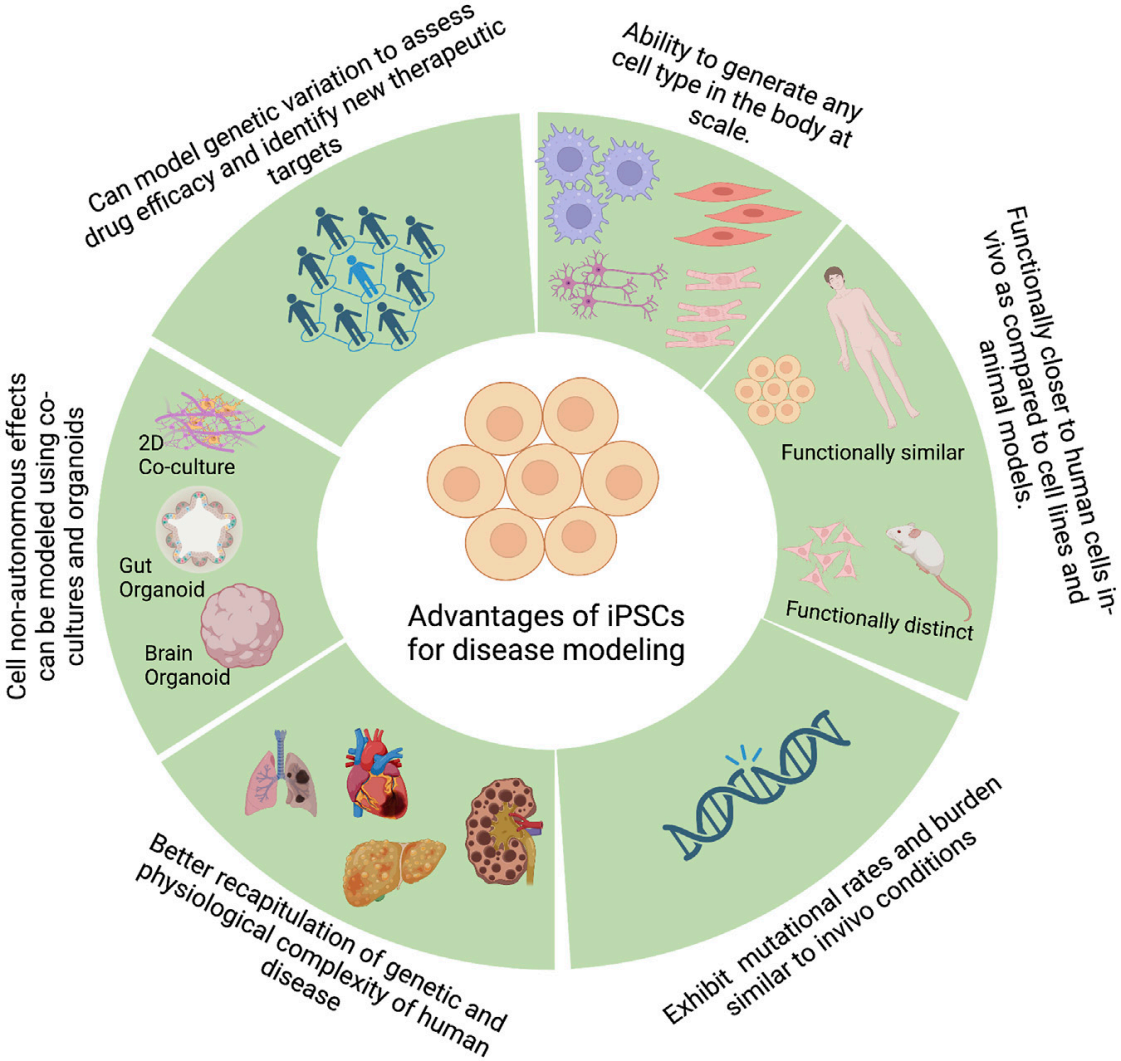
Advantages of pluripotent stem cells for disease modeling. Figure created with BioRender.com.

One of the primary steps during drug discovery and development is identifying the causative genes that lead to a given phenotype or a disease. To effectively identify such causal genes, we need a model system that can accurately recapitulate the molecular phenotypes of the disease and an unbiased method of perturbing genes to study the effect of such perturbations on the disease phenotype. PSCs, by virtue of their broad differentiation potential, have shown great promise in disease modeling by enabling the generation of disease-relevant cell types, among other promising characteristics (Figure 2).

### Advantages of PSCs over immortalized cell lines and primary cells

Traditionally, cell lines and animal models have been widely used for disease modeling, but they present several challenges. Cell lines and/or primary patient-derived cells have been used to model several diseases, including neurological disorders such as Alzheimer’s and Parkinson’s disease (Ferrari et al, 2020; Cetin et al, 2022), immunological disorders rooted in macrophage dysfunction (Daigneault et al, 2010; Chanput et al, 2014; Mendoza-Coronel and Castañón-Arreola, 2016), cystic fibrosis (McCarron et al, 2021), and others. However, some limitations as disease models exist, as outlined below.

1. Primary cells have limited expansion capacity, and certain populations of cells may be difficult to access.
2. Cell lines often require additional differentiation steps to be appropriate model systems, such as retinoic acid differentiation for SH-SY5Y cells (Shipley et al, 2016) or PMA differentiation for THP1 and U937 cells (Wu et al, 1994; Daigneault et al, 2010; Song et al, 2015).
3. The immortalization process can cause phenotypic and functional changes in the cells. For example, it has been shown that NGF treatment causes PC12 cells to differentiate into a neuronal phenotype (Hu et al, 2018), however, they produce unusual combination of neurotransmitters (dopamine, norepinephrine, and acetylcholine) not observed in normal neurons.
4. Most cell lines have an oncogenic origin and/or acquire additional mutations or chromosomal aberrations during the immortalization process and subsequent cell culture. The presence of these mutations might mask the effect of disease-causing mutations.
5. Some of the disease-relevant cell types could be rare cell populations that are difficult to isolate from patients, making it a challenge to convert them into immortalized cell lines.

PSC-derived cells offer advantages in all these aspects. Human embryonic stem cells (hESCs) and iPSCs can be differentiated into all the cell types originating from the three germ layers (Thomson et al, 1998; Reubinoff et al, 2000; Takahashi and Yamanaka, 2006; Takahashi et al, 2007) in addition to trophoblast (Kojima et al, 2017) and yolk-sac derived cells (Atkins et al, 2021).

As noted in point 4 above regarding mutations in cell lines, while PSCs may also manifest with mutations, these can be minimized. Three broad sources lead to accumulation of somatic mutations in iPSCs: pre-existing mutations in the starting somatic cells, reprogramming-induced mutations, and passage-induced mutations, all of which can be avoided. A large proportion of iPSC lines were originally derived from skin fibroblasts, which could explain the higher mutational load in starting somatic cells, as skin cells are exposed to environmental stress and are therefore more prone to acquire mutations. However, iPSCs can also be derived from several other starting cells, such as peripheral blood mononuclear cells (PBMCs) (Loh et al, 2009), cells isolated from urine (Xue et al, 2013), mesenchymal stromal cells derived from wisdom teeth (Oda et al, 2010), or human umbilical vein endothelial cells (Panopoulos et al, 2011). PBMC-derived iPSCs have higher cytogenic stability and lower mutational burden as compared to skin fibroblast-derived iPSCs (Panther et al, 2021). Hence, cell types with lower mutational burden may be an optimal choice to reprogram into iPSCs.

The reprogramming method can affect the number of mutations acquired during the reprogramming process. Studies have shown that the use of non-integrating vectors for iPSC reprogramming minimizes the genomic instabilities compared to the use of integrating vectors such as retroviruses (Kang et al, 2015; Schlaeger et al, 2015; Turinetto et al, 2017). The length of iPSC culture can also affect the acquisition of mutations, which can be limited by reducing culture time (Turinetto et al, 2017) and optimizing culture conditions to reduce oxidative stress (Kuijk et al, 2020).

Recent research comparing mutational burden of iPSCs with isogenic embryonic cells during embryogenesis revealed a similar mutational rate and burden (Hasaart et al, 2022), indicating that iPSCs do not have increased propensity to acquire mutations *in-vitro*. Furthermore, advanced NGS techniques such as whole exome sequencing and whole genome sequencing should be used to estimate the mutational load and prioritize iPSC lines for use in disease modeling and drug discovery.

### Advantages of PSCs over animal models

iPSC-derived cells also offer advantages over animal models of disease. It has been reported that about 92% of drugs found safe and therapeutically effective in animal models fail in clinical trials either due to toxicity or inefficacy (Arrowsmith, 2011; Mak et al, 2014; Seifirad and Haghpanah, 2019). Additionally, more than 90% of existing drugs only work in 30%–50% of people (Roses, 2000). These failures are attributed to the inability of animal models to fully recapitulate human disease phenotypes. For example, studies have shown that gene expression profiles of human autoimmune disease and murine models of autoimmune disease are dissimilar (Liu et al, 2004). Similarly, murine models have been found to be quite inadequate for metabolic disorders like Fabry disease (Ohshima et al, 1997) and Lesch-Nyhan syndrome (Moro and Hanna-Rose, 2020). In contrast, iPSC-derived disease models, being of human origin, have the potential to better recapitulate human disease phenotypes, as observed in cases of complex psychiatric disorders. iPSC-derived cell models of psychiatric disorders offer advantages over animal models in replicating the genetic and physiological complexity of human disease. In addition, they allow for high-throughput neurophysiological assessment of neural networks and biochemical/epigenetic assessment at the cellular and subcellular level. These advantages have been reviewed by Falk et al (Falk et al, 2016).

### Modeling cell non-autonomous effects using co-culture and organoids

Monotypic differentiation systems discussed above are good for modeling cell autonomous disease mechanisms. However, many diseases have non-autonomous multicellular contributions, and the disease relevance of functional genomics screens improves as the screening model becomes more like the cell type(s) affected by the disease. This represents another opportunity for PSC-derived cell model development, as PSCs can be expanded and differentiated prior to co-culture, making sophisticated multi-culture and assembloid-type platforms possible. Co-culture systems, where two or more PSC-derived cell types are cultured together, and organoid cultures, where PSCs are differentiated to self-organize into 3D structures, have shown great promise in studying cell non-autonomous mechanisms of disease. Several recent studies have shown that co-culture systems were critical for identifying disease phenotypes that arise from cell-to-cell communication. Neuron-astrocyte co-cultures have shown astrocyte contributions to neurological disorders such as ALS (Zhao et al, 2020), Parkinson’s Disease (de Rus Jacquet et al, 2021), Alzheimer’s disease (Wasilewski et al, 2022), and epilepsy (Ahtiainen et al, 2021). Co-cultures of iPSC-derived cardiomyocytes and cardiac fibroblasts reverted gene expression and electrophysiological properties to a tissue like state (Beauchamp et al, 2020). Neuron, astrocyte and microglia co-culture models have revealed key astrocyte and microglia functions such as cytokine production and synaptic pruning with implications in neurological disease (Harschnitz, 2019; Sellgren et al, 2019; Baxter et al, 2021).

3D organoid systems offer additional advantages over co-culture systems, as they offer a 3D tissue environment and closely mimic cellular organization seen in tissues. Stachowiak et al, 2017 observed abnormal patterns of proliferation of neural progenitor cells (NPCs) in the ventricular zone, intermediate zone and cortical zone in brain organoids modeling schizophrenia which could not have been observed in 2D culture systems. An iPSC-derived cortical organoid model for 22q11.2 deletion syndrome showed transcriptional and electrophysiology defects in neurons and could also be used as a platform to test antipsychotic drugs which could reverse these defects (Khan et al, 2020). Organoid differentiation protocols have now been developed for tissues originating from all three germ layers (ectoderm, mesoderm, and endoderm) and have shown functional properties, which enable modeling human organogenesis, homeostasis, injury repair and disease [reviewed in (Lancaster and Huch, 2019)]. While these sophisticated models are valuable, it is important to balance model complexity *versus* practicality, as co-culture or organoid systems are often able to do a better job of recapitulating complex disease biology but may be intractable as large-scale screening systems (Durens et al, 2020).

## Establishing relevant and robust PSC models

### Identifying relevant cell lines

There are several important factors to consider when developing a PSC-derived model, the first of which should be selecting an appropriate cell line or cohort of cell lines. Several repositories exist with patient derived iPSCs from different genetic backgrounds which can serve as valuable models with which to study disease. In addition to genetic background, these cell lines include information on the donors’ age, sex, ethnicity and health status, factors which can further increase the relevance of a disease model (Warren and Cowan, 2018). Depending on the scope of the experiment however, a large cohort of disease relevant iPSC lines may be required. For example, an iPSC-derived screen to uncover disease-associated quantitative trait loci (QTLs) or genome wide association studies (GWAS) may require a cohort large enough to study subtle phenotypic variation at specific loci. In such cases, recruitment of tissue donors may be necessary if there are not enough existing iPSC lines to adequately power the study. Due to the considerable cost associated with generating new iPSC lines, several organizations such as the Next-Generation Genetic Association Studies (NextGen) Consortium and Stanford Cardiovascular Institute Biobank are endeavoring to widen the number of disease relevant iPSC lines available to researchers (Musunuru, 2018).

### Optimizing differentiation protocols

Of equal importance to gathering relevant iPSC lines is developing a robust and scalable differentiation protocol to a cell type of interest. To this end, gaining a good understanding of the range of differentiation efficiencies to expect by first working in model iPSC lines can aid in prediction of phenotypes that may be encountered in a cohort of experimental iPSC lines. It is also important to make sure that the cell culture requirements necessary to achieve the desired cell type are amenable to the overall experimental design, and in the case of larger population modeling cohorts, is scalable. Finally, establishing differentiation benchmarking metrics, for example, by cell marker expression or functional profiling, is required to ensure that the resultant cell type faithfully represents the phenotypes of interest to the study (Quadrato et al, 2016).

### Establishing adequate cell line controls

Another consideration when designing an iPSC-derived model is the selection of appropriate controls to isolate genotype-to-disease relationships. When modeling a monogenic disease, this is often accomplished by engineering an isogenic control line using CRISPR-Cas9 to knock in or out the causal gene variant within the same iPSC genetic background (Bassett, 2017). This method is straightforward, however, with a larger cohort of iPSCs it becomes difficult to generate isogenic pairs for each unique donor line. In instances like this with large populations of donor lines, there is a degree of built in locus-specific control as genomic regions in each unique iPSC line can act as controls to one another. This form of locus-specific control is appropriate for use in GWAS or QTL based studies, however the gold standard would still be generation of isogenic controls. When attempting to model more complex polygenic, or highly penetrant diseases, the engineering challenge is greater for establishing iPSC line controls. However, if the causal genes associated with the disease being modeled are well known, the possibility of making multiple gene perturbations simultaneously in the same cell has been shown to be effective (González et al, 2014) and could allow for the generation of isogenic controls for more complex diseases.

### Modeling the effects of genetic variation using iPSCs

Apart from the functional advantages that PSC-derived model systems offer over other disease models, a unique potential that makes PSCs and PSC-derived cells stand out is the ability to model human genetic variation. Studies profiling transcriptomes of hundreds of iPSCs have revealed that genetic background exerts a larger effect on the variation in resultant iPSC lines than any other non-genetic factor such as culture conditions, passage, gender, etc. (Burrows et al, 2016; Carcamo-Orive et al, 2017; DeBoever et al, 2017; Kilpinen et al, 2017). Since iPSCs can theoretically be differentiated into any cell type of choice, researchers can investigate the effect of genetic variation in the cell type of interest based on the disease. DeBoever et al profiled 215 human iPSC lines and showed that it is possible to examine rare inherited variants (CNVs and SNVs) with moderate effect sizes in iPSCs. This is not possible in animal cell models or patient derived cell models where large numbers of rare cell types may be required. As such, genetically diverse iPSCs can be usefully applied to the three cases outlined below.

First, to examine the effect of a particular pharmacological perturbation on multiple genetic backgrounds. Some ethnicities are underrepresented in clinical trials and conducting pharmacological screens on genetically diverse populations of iPSCs and/or iPSC-derived cells might give insights about drug efficacy and toxicity for such populations before a drug is released into the market. A recent study performed a population-based toxicity screen in iPSC-derived cardiomyocytes and neurons generated from an iPSC bank containing 13 homozygous HLA haplotypes, representing 16% of the population in Taiwan and estimated to represent at least 477,611,135 people in the world (Huang et al, 2022). This group reported inter-individual differences in cardiotoxicity and neurotoxicity for the tested compounds, highlighting the utility of such population-based screens for testing drug toxicity and efficacy. Second, the study of protective or pathogenic variants should be done on multiple genetic backgrounds as this can facilitate assessment of modifier alleles which influence phenotypes leading to novel insights into disease biology. Third, large genetically diverse iPSC cohorts can be used to identify cell type specific eQTL (expression quantitative trait loci) phenotypes. Two different studies identified tissue-specific eQTLs in iPSC-derived hepatocyte-like cells (Pashos et al, 2017; Warren et al, 2017). Pashos et al also performed mechanistic validations to identify causal variants, highlighting the power of this approach to accurately identify causal variants of a phenotype. Such population modeling approaches utilizing large iPSC cohorts can be particularly useful in cases where *in vivo* tissues/cell types are hard to access. Practical considerations for when and how to design experiments with large iPSC cohorts were reviewed recently (Warren and Cowan, 2018). Population based approaches also require the creation of large iPSC banks which contain iPSCs representing different ethnic backgrounds (Ghosh et al, 2022). While Caucasian populations have the most representation in almost all the major iPSC collections, efforts are now being made to improve the representation from other ethnicities. Table 1 lists publicly available iPSC banks along with their ethnic representations. Such iPSC-derived disease models can further be used in unbiased chemical and/or genetic screens to identify disease mechanisms and new therapeutic targets.

**TABLE 1:** Currently available iPSC banks with ethnicity information.

Repository name	Total number of iPSC lines	Ethnicities reported	Hyperlink
CIRM	1554	African American (4.5%), American Indian (0.19%), Asian (6.11%), Asiatic Indian (0.64%), Black (0.58%), Caucasian (70.8%), Chinese (0.38%), East Indian (0.25%), Filipino (0.77%), Hispanic/Latino (5.01%), Japanese (0.25%), Korean (0.19%), Pacific Islander (0.51%), Taiwanese (0.06%), Multiple races (1.7%), Other (7.4%)	https://www.fujifilmcdi.com/search-cirm/
EBISC	949	Caucasian (9.06%), African American (4.21%), Asian (9.16%), Indian (0.5%), Chinese (0.63%)	https://ebisc.org/
CORIELL	218	White (49%), Black/African American (7.79%), Asian (4.12%), Hispanic/Latino (0.45%), American Indian/Alaska Native (1.3%), Not Reported (4.5%), More than one race (1.8%), Unknown (3.2%)	https://www.coriell.org/1/Browse/Biobanks
iPSCORE	222	N/A	http://frazer.ucsd.edu/charvar.html
HipSci	946	European (67.12%), South Asian (3.17%), Ad Mix American (2.74%), African (0.84%), East Asian (0.21%)	https://www.hipsci.org
WiCell	1427	African American (15.06%), Arab (0.84%), Asian (12.12%), Caucasian (65.1%), Latino (5.25%), Native American (0.35%), Pacific Islander (0.07%), Unknown (0.98%)	https://www.wicell.org/home/home.cmsx
CiRA	38	Japanese population only	https://www.cira-foundation.or.jp/e/research-institution/ips-stock-project/
KSCB	50	Korean population	https://nih.go.kr/eng/main/contents.do?menuNo=200061
RIKEN-BRC	15	N/A	https://cell.brc.riken.jp/en/hps
REPROCELL	9	Asian Indian (55.5%), Caucasian (22.2%), Hispanic (11.1%), Filipino (11.1%)	https://www.reprocell.com/product-catalog/induced-pluripotent-stem-cells

## Types of high-throughput screens and examples performed in PSC-derived cells

### High-throughput drug screening

Due to their disease relevance and diverse human origin, hiPSC-based disease models have increasingly been used in the study of disease mechanisms and the development of effective disease-modifying therapeutic targets. The first hiPSC large-scale drug screening was conducted in 2009 by Lee et al, 2009 in familial dysautonomia (FD) patient-specific hiPSCs and led to discovery of the role kinetin plant hormone plays in reducing the pathological phenotype of FD (Lee et al, 2009). Since then, several hiPSC based drug screens have been successfully conducted in various iPSC-derived cell types such as cardiomyocytes, hepatocytes, different disease relevant neurons and neuron progenitors [list summarized in (De Masi et al, 2020)]. Some of the reasons to use iPSC-derived disease models for developing novel drugs include:

1. Lack of appropriate disease relevant cell or animal models that can recapitulate all the disease-specific symptoms
2. When access to the affected cells and pathogenic sites is limited, for example, with neurological and psychiatric diseases
3. iPSCs may be used as biomarkers of disease progression and to understand the effects of therapeutic targets
4. Patient derived iPSCs can be used to estimate the efficacy and safety of the drugs prior to administration

In 2017, Kondo et al, 2017 screened a 1258-pharmaceutical compound library in 13 iPSC-derived neurons from Alzheimer (AD) patients with outputs including amyloid β peptide-Aβ40 and Aβ42 secretion and the Aβ42/40 ratio. After two rounds of screening, 27 Aβ-lowering screen hits were shortlisted, of which 6 lead compounds were further prioritized due to their capacity to reduce Aβ40 and Aβ42 levels in most of the 13 sets of AD neurons. Finally, a combination of three compounds, bromocriptine, cromolyn and topiramate were identified to reduce the Aβ42/40 ratio in iPSC-derived neurons from patients with familial AD but not with sporadic AD. In recent years, targeting Aβ aggregates as the major therapeutic target of AD has been re-evaluated and new therapeutic targets are being investigated.

Another example of an iPSC-derived neuronal drug screen is a 3000-compound screen conducted in human neural stem cells and iPSC-derived neurons from children affected with Lesch-Nyhan disease (LND) (Ruillier et al, 2020). LND is a rare monogenic disease caused by deficiency of hypoxanthine-guanine phosphoribosyl transferase (HGPRT) enzyme and characterized by severe neuropsychiatric symptoms that currently cannot be recapitulated in HGPRT-deficient animal models. This screen identified six pharmacological compounds, all possessing an adenosine moiety, that corrected HGPRT deficiency associated neuronal phenotypes of LND.

Amyotrophic lateral sclerosis (ALS) is a neurodegenerative disease characterized by loss of upper and/or lower motor neurons. Ropinirole, Retigabine, and Bosutinib are three candidate anti-ALS drugs that have been identified in three separate iPSC-derived motor neuron-based drug screens (Wainger et al, 2014; Imamura et al, 2017; Fujimori et al, 2018) and are currently being investigated in clinical trials for safety and effectiveness (Okano et al, 2020).

### CRISPR screening

Modern functional genomics screening strategies generally involve CRISPR/Cas9-based tools including CRISPR nuclease, CRISPRi, and CRISPRa (Gilbert et al, 2014; Shalem et al, 2014), modalities that involve different considerations around cell engineering and delivery and readout strategies. CRISPRi and CRISPRa use a catalytically inactive form of Cas9 (dCas9) fused to transcriptionally repressive or activating domains, respectively. Both CRISPR nuclease and CRISPRi allow for testing of the effects of loss of gene expression, but they differ in important ways. CRISPR nuclease cleaves the gene of interest so is irreversible and does not require sustained expression of Cas9 machinery, but can result in truncated or hypomorphic proteins depending on the cut site (Michlits et al, 2017). In addition, it can be delivered recombinantly conjugated with a gRNA via electroporation in the form of a ribonucleoprotein (RNP) complex (Liu et al, 2015), allowing for greater flexibility in delivery mechanism. CRISPRi is transiently active and reversible so most applications require establishment of a cell line with stable expression of the dCas9 fusion protein to maintain repression over time. The repression induced by CRISPRi is generally more uniform across a population *versus* with CRISPR nuclease but may not be a complete loss of expression depending on the cell type and locus. This lack of complete repression can also be a benefit when the goal of the screen is to mimic the effects of small molecule therapeutics to identify candidate gene targets. CRISPRa screens can provide complementary information to CRISPRi screens, where genes are down- or upregulated at endogenous loci and hits can be compared to identify common hits that are enriched in opposing populations with the two screening modalities.

Other domains that have been fused to elicit different effects on gene regulation include CRISPRoff/on (Nuñez et al, 2021) and other epigenetic editors that have been reviewed elsewhere (Nakamura et al, 2021). Other forms of Cas proteins provide other advantages such as the ability to perturb multiple genes simultaneously with Cas12a, and compact size for efficient packaging and delivery with Cas12f (Wu et al, 2021; Xu et al, 2021).

**FIGURE 3:**
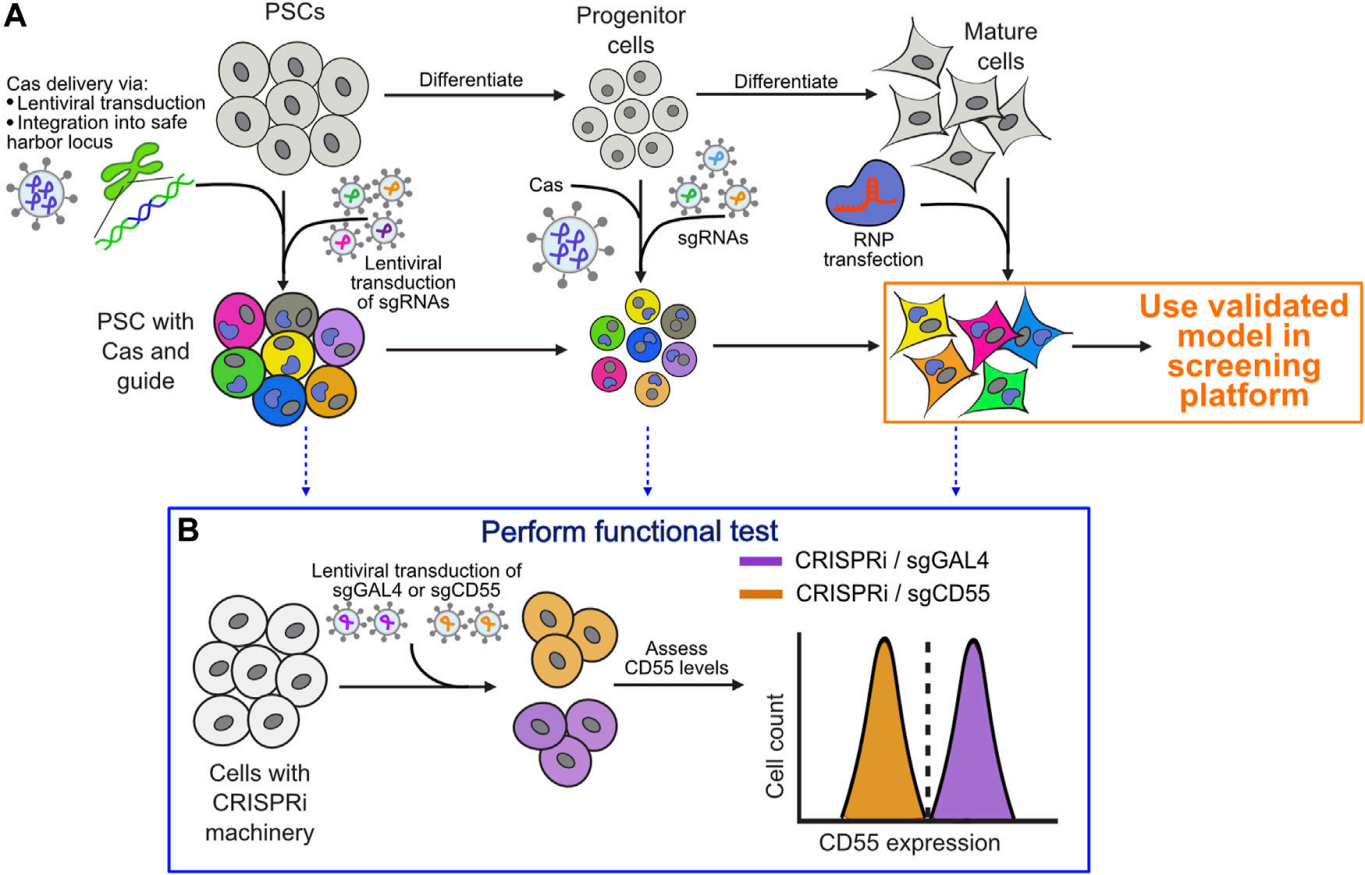
Considerations for when and how to introduce CRISPR/Cas9 transgenes into iPSC-derived cells for downstream screening purposes. **(A)** Progression of differentiation from pluripotent to differentiated state and preferred methods to deliver Cas machinery and guides at each stage. Functional testing should be performed after introducing guides and machinery and at the final stage of differentiation prior to using cells in a screening platform. **(B)** Example functional testing workflow using the cell surface protein CD55 with a flow cytometry-based readout to test functionality of CRISPRi machinery. A similar approach can be used to assess gene knockout or overexpression.

CRISPR screens require expression of Cas9 machinery as well as the guide RNA targeting the gene of interest, which can be expressed on the same or different constructs. Most screens using iPSC-derived cells with stable expression of CRISPR/Cas9 machinery involve generation of iPSCs with genetically integrated Cas9 machinery and lentivirally expressed guide RNAs (Figure 3A). Alternatively, RNP-based delivery can be more approachable for many cell types but is not suitable for pooled screening unless guides are expressed stably. Likewise, a transposon-based system such as piggyBac can be used for more stable expression of individual and multiplexed sgRNAs, though they have been shown to cause insertional mutagenesis (Li et al, 2017). For stable expression of machinery, options include expression from a safe harbor locus or lentiviral expression. Expression in a targeted safe harbor locus can help prevent silencing of expression machinery over time or upon differentiation (Sadelain et al, 2012), but requires more sophisticated cell engineering techniques and longer timelines for cell line generation as well as clonal selection in the case of PSCs. Lentiviral-mediated expression is simple to implement, and if the construct contains a resistance or fluorescent selection marker then the resulting population can often be used as a pool, thereby limiting concerns about artifacts based on genetic integration site. However, it is crucial to ensure that the CRISPR machinery is expressed and functional on a population-wide level in the differentiated cell population that will be used in the screen, which can be tested using flow cytometry with a guide targeting a cell surface protein. In this assay, a guide targeting a well-expressed but non-essential cell surface protein is stably introduced and cells are analyzed by flow cytometry to verify that every guide-expressing cell has active machinery (Figure 3B).

## Development of screening assays and readouts

**FIGURE 4:**
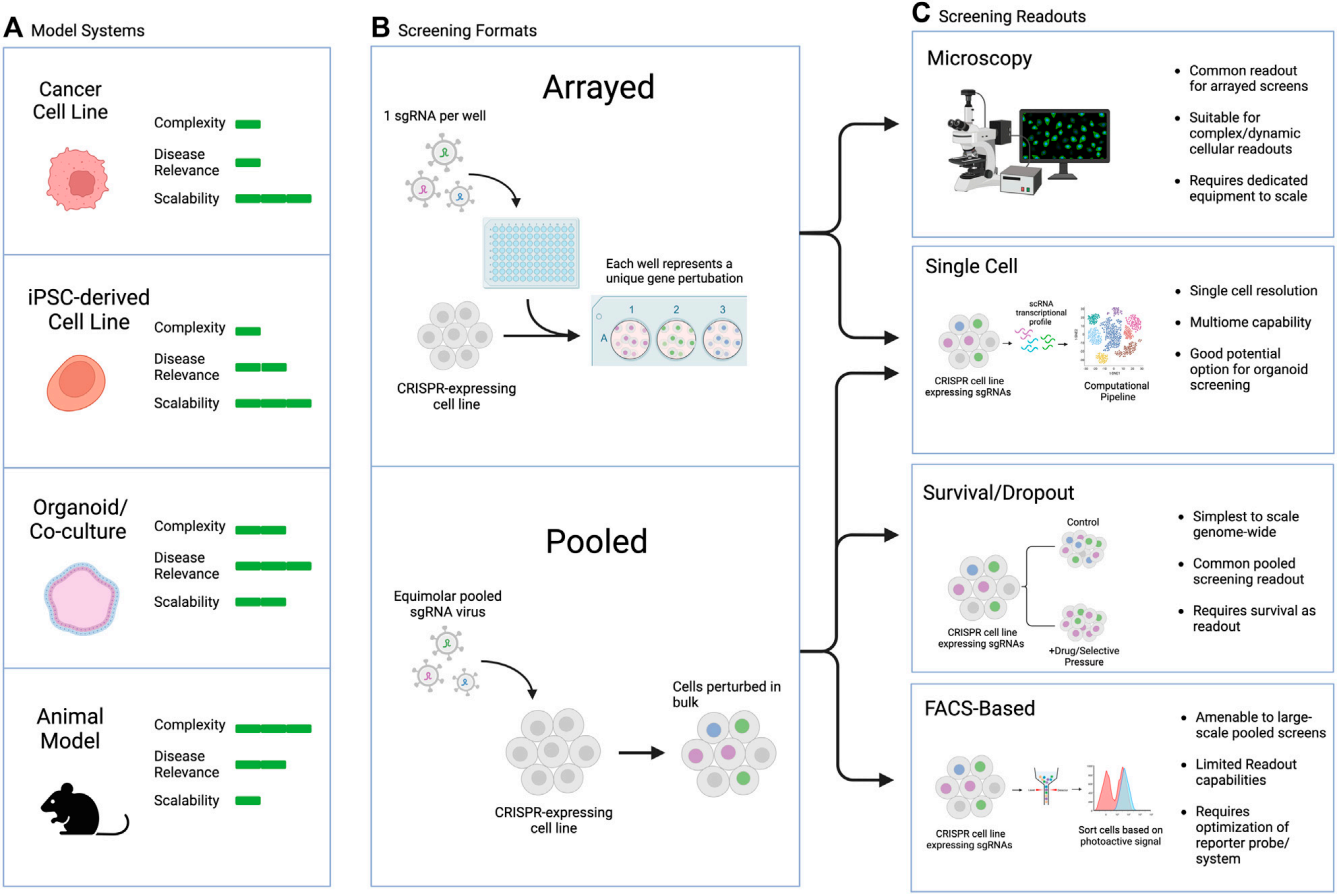
Commonly used CRISPR screening readouts and applicability to PSC-based screens. **(A)** Model selection considerations, including pros and cons of each model type. **(B)** General types of screens discussed in this review. **(C)** Screening readouts including pros and cons of each assay type and their applicability to different screening modalities.

Large-scale screens can involve different types of manipulations, from screening across small molecule libraries to genetic screens using siRNA, cDNA-based overexpression, or CRISPR guide libraries, among other possibilities. The readouts can also range broadly, with two main categories: arrayed screens, which are generally performed in multi-well plates with different perturbations tracked in each well, and pooled screens, which are generally performed in large culture vessels with all perturbations combined and read out downstream (Figure 4). Generally, survival or FACS-based pooled systems are more amenable to large-scale screens, including genome-wide perturbation, but they suffer from limited readout capabilities and require significant upfront optimization to ensure the dynamic range is appropriate to capture the biology of interest. On the other hand, arrayed screens are more difficult to scale as they require individual wells for each perturbation of interest, but they are suitable for complex or dynamic intercellular readouts, rendering them more flexible in terms of assay design. Combining the strengths of both approaches in pooled optical screening platforms is an exciting new avenue that could hold promise for screens in iPSC-derived complex models (Feldman et al, 2019; 2022).

The appropriate readout and assay to use for a screen depends on the biological question of interest. Typical pooled screen readouts include survival/dropout of cells over time, FACS-based sorting or selection of a dye, protein, or reporter of interest, or single cell sequencing readouts (Przybyla and Gilbert, 2022). Arrayed screen readouts are generally microscopy-based or utilize a plate reader for ELISA, luminescence, or fluorescent endpoints. In addition to biological question, the choice of endpoint may also depend on the screening model chosen. For example, PSC-derived organoids are amenable to single cell readouts, particularly in the context of genetic CRISPR-based screens. This approach can allow for simultaneous elucidation of cell type, cell state, and genetic perturbation, which can be especially useful for complex heterogenous organoids or assembloids consisting of several different cell types.

The utility of PSC-derived cells for disease modeling requires identification and optimization of differentiation protocols to generate the relevant cell type, as described above. Equally important is adequate testing to ensure that the cellular phenotype and/or function of interest is exhibited. Examples of cases where this has been applied include iPSC-derived macrophages, which express macrophage markers, secrete pro-inflammatory and anti-inflammatory cytokines, and exhibit phagocytic activity (Lachmann et al, 2015; Nenasheva et al, 2020; Lyadova and Vasiliev, 2022). IPSC-derived microglia have been shown to display characteristics of primary microglia including ability to phagocytose neuronal debris (Andreone et al, 2020). IPSC-derived neurons show capacity to form mature synapses (Meijer et al, 2019), release neurotransmitters (Hook et al, 2014) and fire action potentials (Prè et al, 2014).

Once an assay and readout are chosen, they need to be optimized and validated to ensure suitability for large-scale functional genomics screening applications. The specifics will depend on the assay, but, in general, it is important to ensure that an assay provides a sufficient dynamic range to capture the signal of interest above noise for the largest possible perturbation window. If the assay involves a stimulus and subsequent readout (e.g., survival, flow cytometry, microscopy), then it is important to ensure that the stimulated condition is clearly separated from the control with minimal signal overlap. For genetic validation, it can be helpful to test manipulate some control genes that are expected to demonstrate specific phenotypes in your assay to ensure they give the appropriate phenotypic shift, which provides greater confidence that the screen will adequately capture the biology of interest.

The final step in a screening workflow is analysis and hit calling, which again will vary based on assay and readout. The available analysis tools for pooled screens are described and summarized elsewhere (Doench, 2018; Hanna and Doench, 2020), but in general involve collecting the selected/sorted cell populations, extracting the genomic DNA, PCR-amplifying the guides represented, and sequencing to identify the enriched guides in each population over the relevant control. Arrayed screens require normalization strategies across plates and wells to minimize batch effects and artifacts, in addition to image analysis pipelines in the case of microscopy-based screens.

## Applications of CRISPR/Cas screening in hiPSC-derived cell types

Large-scale functional genomics screens implementing iPSC models have been used across several disease areas to gain insights into molecular mechanisms underlying disease biology and to identify new therapeutic targets or pathways of interest. Several cell types and disease areas are highlighted below. (Li et al, 2019; Guo W. et al, 2022).

### Cardiomyocytes

Doxorubicin is a common chemotherapy compound used to treat various cancers but results in severe side effects, including heart failure. A genome wide pooled CRISPR KO screen in iPSC-derived cardiomyocytes was performed and identified two new targets, SLCO1A2 and SLCO1B3, which are both human-specific transporters whose loss of function protects cardiomyocytes against doxorubicin-cardiotoxicity but does not affect cell death in cancer cells (Sapp et al, 2021).

### Human stem cell-derived islets

Stem cell-derived islets generated by directed differentiation from hPSCs are a great source for pancreatic β cell replacement therapy to treat insulin-dependent diabetes. In recent years there has been promising clinical trial data using progenitor cells (Ramzy et al, 2021) or fully differentiated and functional stem cell (SC)-islets (Vertex, 2021). However, the major challenge of protecting SC-islets from an immune response remains. To understand the underlying pathways that drive immunogenicity of SC-islets in inflammatory environments, Sintov et al performed single-cell RNA sequencing and whole-genome CRISPR screen of SC-islets under immune interaction with allogeneic peripheral blood mononuclear cells (Sintov, 2022). The screen results indicated that targeting the JAK/STAT type II interferon pathway by depleting chemokine ligand 10 (CXCL10) will provide reduction of SC-islet immunogenicity.

### Neurons

Examples of screens performed in the context of neuronal disease include a genome-wide pooled CRISPR-Cas9 knockout (KO) screen in human neural progenitors to identify molecular therapeutic targets that disrupt the host-dependent mechanism of Zika virus infection (Li et al, 2019). Another recent example is a kinome-wide (sgRNAs against 736 kinases) survival-based KO screen conducted in hiPSC-derived cortical neurons to identify modifiers of poly (PR) dipeptide repeat protein toxicity, the most common genetic cause of frontotemporal dementia (FTD) and amyotrophic lateral sclerosis (ALS) neurodegenerative diseases (Guo W. et al, 2022). The screen identified NEK6 as a novel therapeutic target for C9orf72-related FTD or ALS, which regulates poly (PR)-mediated p53-related DNA damage.

Some of the challenges with traditional neuronal differentiation methods include multi-step protocols that take several weeks, can be difficult to scale up, and usually yield heterogeneous populations of differentiated cells. One promising alternate method for hiPSC-derived differentiation, however, is transcription factor-directed differentiation of hiPSCs. For example, overexpression of neurogenin 2 (NGN2) transcription factor from AAVS1 safe-harbor locus in hiPSCs directs the differentiation into glutamatergic neurons with high efficiency and homogeneity (Zhang et al, 2013; Wang et al, 2017; Fernandopulle et al, 2018). Tian et al, 2019 utilized Ngn2-driven generation of glutamatergic neurons and performed two CRISPRi/a-based screens with survival or FACS-based phenotypes. In the first study, they revealed neuron-specific essential genes and genes that improved neuronal survival upon knockdown, while in another study the authors identified a novel link between lysosomal failure to ferroptosis in human neurons by knockdown of the lysosomal protein prosaposin (Tian et al, 2021).

### Microglia

Another example of transcription factor-directed differentiation of hiPSCs is generation of microglia-like cells based on the inducible expression of six transcription factors in hiPSCs following an 8-day efficient protocol (Dräger et al, 2022). In this study, inducible CRISPRi and inducible CRISPRa screens were performed against the druggable genome in transcription factor-directed hiPSC-derived microglia, uncovering insights into microglial biology including genes controlling survival, activation, and phagocytosis. The screens identified *PFN1* and *INPP5D* as novel modulators of phagocytosis in microglia, and variants in these genes can possibly be associated with neurogenerative diseases.

### Astrocytes

Leng et al performed a pooled CRISPRi screen in hiPSC-derived cells, identifying cellular pathways that control cytokine-induced inflammatory astrocyte reactivity (Leng et al, 2022). To generate hiPSC-derived astrocytes in a scalable manner, Tcw et al, 2017 used a modified protocol by overexpressing gliogenic transcription factors NFIA and SOX9 during the differentiation process, as described previously (Li X. et al, 2018). These results and the scalable hiPSC-derived astrocytes platform have the potential to guide the development of therapeutics to selectively modulate different aspects of inflammatory astrocyte reactivity.

## Future directions

The rise of CRISPR-based screening techniques over the past decade has drastically increased the power of genetic screening to probe biological questions across a wide range of models. We anticipate that CRISPR screening in iPSC-derived models will expand this capability by allowing for the ability to interrogate disease biology in more relevant contexts. In addition to the CRISPR screening systems discussed in this review, there are several new CRISPR modalities and emerging screening technologies that have the potential to further drive biological discovery.

Of particular interest is the emergence of epigenetic remodeling systems that not only offer the ability to cause reversible epigenome-level silencing but could allow us to study how epigenomic regulation plays a role in disease biology and cell identity. One such system is CRISPRoff/on, which deposits targeted methylation marks via its DNMT3A/3L methyltransferase domains and can likewise reverse this modification with the demethylase activity of TET1. Through transient expression of the CRISPRoff system, robust epigenetic silencing can be achieved and has been shown to be maintained in iPSCs through differentiation (Nuñez et al, 2021). Similar systems such as the “hit-and-run” approach have shown that even longer-term epigenomic silencing can be achieved by the combinatorial targeting of DNMT3A/3L and the histone methyltransferase Ezh2 which promotes prolonged DNA methylation by silencing H3K27ac (O’Geen et al, 2019). Epigenetic remodelers such as CRISPRoff/on could offer the unique ability to perform screens to interrogate the role the chromatin landscape plays in specific diseases and uncover important cell-state transition biology, as it is becoming increasingly apparent that chromosome organization is largely cell type-specific. Additionally, the transient and reversable nature of these systems make them promising for potential therapeutic applications.

Other capabilities that show future promise involve screens in complex organoid and co-culture models, however, there are several current limitations that need to be overcome for these systems to substantially reduce the need for animal experiments, as outlined in a prior review (Kim et al, 2020). Organoids can be used for basic research applications to study developmental processes, cell-cell interactions, or response to external stimuli; for disease modeling similar to approaches described here that utilize patient-derived iPSCs; or for precision medicine where patient-derived organoids could be used to understand patient-specific response to drugs. Some of the pros of organoid-based models are that they allow us to bridge the gap between animal models and humans, they can utilize the gene editing tools that have already been developed for other PSC-based systems, and in many cases, they can be scaled up relatively easily in the context of therapeutic or functional genomics screening. However, a major barrier of wider adoption or systematic use of these models is intra-organoid variability, wherein there is a lack of widely accepted standardized protocols. Single cell profiling analysis of the transcriptome and epigenome of organoids might help overcome this issue by allowing for comparison of the heterogenous cell types with their *in vivo* counterparts.

The rapid increase in the number of GWAS has led to the identification of numerous single nucleotide polymorphisms (SNPs) and eQTLs associated with a variety of disorders. Most eQTLs are identified using expression data from bulk tissue samples or easily accessible cell types, but numerous studies have shown that a considerable proportion of eQTLs are cell-specific, tissue-specific and sometimes even specific for a particular region in the tissue (Hovatta et al, 2007; Majumdar et al, 2021; Bryois et al, 2022).This presents a unique opportunity to use iPSCs and iPSC-derived, disease relevant cell types to accurately identify cell type specific eQTLs. Recent studies using iPSC-derived cells have shown great promise in identification of cell type-specific eQTLs (Strober et al, 2019; Neavin et al, 2021; Elorbany et al, 2022). One such study used 125 donor iPSC lines and identified hundreds of eQTLs that change during endoderm differentiation via single cell RNA sequencing (Cuomo et al, 2020). Another study that explored this idea of *in vitro* population genetics used 68 iPSC lines to identify metabolic and transcriptomic phenotypes of a SNP for metabolic disease (Warren et al, 2017). Future studies could use larger iPSC cohorts from genetically diverse populations in complex iPSC differentiation protocols to accurately understand contributions of genetic variation to disease.

An additional application of GWAS data is to design experiments that would functionally validate disease-associated eQTL and SNP loci to identify the disease-relevant target genes. This is complicated by the fact that the majority of the disease-associated eQTL and SNP loci lie in non-coding regions of the genome, which can regulate their target genes over long genomic distances. A few CRISPR based approaches have shown immense promise in accurately mapping enhancers to their target genes. In one such experiment the authors developed CRISPRi-FlowFISH where the gRNAs were targeted to cis-regulatory elements and the effect on expression of target genes was assessed using RNA FISH (Fulco et al, 2019). The authors designed a pooled assay where they sorted cells based on the FISH signal and sequenced gRNAs from the sorted populations to infer if the cis-regulatory element resulted in increased or decreased expression of the target gene (Fulco et al, 2019). Genome-wide mapping of cis-regulatory elements to their target genes is also possible as shown in recent studies (Gasperini et al, 2019; Chardon et al, 2023) that used multiplexed CRISPRi/a perturbations followed by scRNAseq. Future applications of this technology could involve use of complex 2D and 3D iPSC-derived models and CRISPR based perturbations to identify cell type specific cis-regulatory loci enriched for disease eQTLs/SNPs. Improvement in the accuracy of base editing/prime editing systems can also help in engineering of disease associated SNPs in iPSC-derived disease relevant cells to directly infer the function of these SNPs.

While this review focused largely on Cas9-based CRISPR systems, other Cas enzymes provide additional opportunities, including Cas12a which is more amenable to multiplexing due to the mechanism by which its gRNA arrays are processed. Recent advances in dCas12a approaches for transcriptionally modulating genes without introducing double stranded breaks will allow for more sophisticated studies of pathway redundancy and protein cooperativity (Guo L. Y. et al, 2022). For PSCs and differentiated cell types, this could be useful for studying developmental fate decisions and to tease apart molecular pathway components in terms of necessity *versus* sufficiency. In addition, the ability to robustly utilize multiple Cas variants in PSCs would allow for multiplexing across modalities, for example, combining a dCas9-CRISPRa system with a dCas12a-CRISPRi system. This type of parallel multiplexing could be used, for example, to dynamically modulate transcription factor up- and downregulation to understand differentiation pathways and deterministically drive differentiation toward lineages of interest.

Recent advances in iPSC model systems combined with our rapidly expanding databases of human genetic and genomic data and our ability to functionally perturb genes and non-coding regions of the genome have allowed for incredible advances in our understanding of human disease biology. Researchers have uncovered novel mechanisms underlying disease predisposition, initiation, and progression and, in doing so, have identified new therapeutic targets. As these fields continue to co-evolve it will be exciting to see how they combine in new ways with new models, screens, and readouts to continue to drive our understanding of human development and disease.
